# Synergistic Proliferation Effects of Xanthohumol and Niflumic Acid on Merkel and Glioblastoma Cancer Cells: Role of Cell Membrane Interactions

**DOI:** 10.3390/ijms252011015

**Published:** 2024-10-13

**Authors:** Monika Stompor-Gorący, Aleksandra Włoch, Priti Sengupta, Anna Nasulewicz-Goldeman, Joanna Wietrzyk

**Affiliations:** 1 Department of Pathophysiology, Institute of Medical Sciences, University of Rzeszów, Warzywna 1a, 35-310 Rzeszów, Poland; 2 Department of Physics and Biophysics, Wrocław University of Environmental and Life Sciences, 50-375 Wrocław, Poland; aleksandra.wloch@upwr.edu.pl (A.W.); pritisengupta1@gmail.com (P.S.); 3 Department of Experimental Oncology, Hirszfeld Institute of Immunology and Experimental Therapy, Polish Academy of Sciences, Weigla 12, 53-114 Wrocław, Poland; anna.nasulewicz-goldeman@hirszfeld.pl (A.N.-G.); joanna.wietrzyk@hirszfeld.pl (J.W.)

**Keywords:** xanthohumol, niflumic acid, Merkel cancer, glioblastoma, MIMIC

## Abstract

The objective of our research was to determine the effects of xanthohumol (XN), a flavonoid isolated from hops (*Humulus lupulus*), and the anti-inflammatory drug niflumic acid (NA), separately and in combination with each other, on the proliferation of human cancer cells. Additionally, so as to understand the mechanism underlying the anticancer properties of the tested compounds, their effects on the biophysical parameters of a model membrane were assessed. The cells were incubated with XN and NA at various concentrations, either individually or in combination with each other. Cell proliferation was quantified using the sulforodamine B (SRB) assay. In addition, the IC_50_ values for niflumic acid and xanthohumol applied separately were determined by cell proliferation tests for the following human cancer cell lines: 5637 (urinary bladder carcinoma), A-431 (epidermoid carcinoma), UM-SCC-17A (head and neck squamous carcinoma), SK-MEL-3 (melanoma), MCC13 (Merkel cell cancer), and A172 (glioblastoma), in comparison with the mouse normal fibroblasts (BALB/3T3 clone A31). The results show that the two-compound combinations of XN and NA significantly decreased the proliferation of cancer cells in a dose-dependent manner, and the effects were stronger than the additive responses to XN and NA individually. The membrane studies revealed a synergistic effect on the membrane rigidity when using the mixture of XN and NA, which may explain the observed increase in anticancer activity for the combined XN and NA. Our results suggest that NSAIDs, such as niflumic acid, may be a promising strategy for co-application with xanthohumol as anticancer drugs.

## 1. Introduction

Xanthohumol (XN), being one of the main active substances found in common hop (*Humulus lupulus*), has multiple pharmacological activities [1,2]. It belongs to the group of polyphenols with anti-inflammatory and antimicrobial properties [3,4]. The research carried out so far has shown that xanthohumol has cytotoxic activity against a range of cancer cells, including colon [5], breast [6], ovarian, bladder, nasopharyngeal [7], glioblastoma [8], melanoma [9], neuroblastoma [10], colon and hepatocellular [11], human leukemia [12], and gastric cancer cells [13], acting by various mechanisms and signaling pathways [14,15,16,17].

Despite the discovered and well-documented beneficial properties of xanthohumol, its practical application is limited, mainly due to its low physiological availability, but also because of the low yield of its total synthesis, which is only 10% [18]. In order to enhance its therapeutic activity, there are multidirectional studies that have been carried out to obtain new xanthohumol derivatives with new biological activities [19,20,21], as well as to develop new delivery systems for XN, based on nanocarriers [22,23,24]. One of the effective ways of XN delivery involves using thermodynamically stable sophorolipid-based microemulsions [25]. Good effects were also achieved in the pharmacodynamic analysis of a guar gum–pectin-based formulation of xanthohumol [26].

Although there are many research reports on derivatives of therapeutic substances with high application potential, the time-consuming and costly procedures associated with clinical trials, which are necessary for their implementation and registration, have prompted researchers to look for new applications of already used therapeutic agents, which are known to be safe. The molecular-level interactions of xanthohumol and its derivatives with known drugs, as well as with other active substances, have also been studied [27,28,29,30,31,32]. It has been proved that XN used in co-therapy with interferon alpha-2B enhances the antiviral effect of this drug, commonly used to treat chronic hepatitis B and C [33]. Additionally, due to its antioxidant properties, XN has a protective effect on hepatic cells exposed to acetaminophen [34]. Xanthohumol and its derivatives also showed anti-inflammatory activity, and the initial results of the clinical trials indicated that XN has the potential to be used for the treatment of immune-related diseases, such as Crohn’s disease [35] and allergic skin diseases, such as chronic allergic contact dermatitis [36].

In order to relieve pain associated with diseases, e.g., cancer-related pain, non-steroidal anti-inflammatory drugs (NSAIDs) are often used, such as fenamic acid derivatives, including mefenamic acid, niflumic acid, meclofenamic acid, and flufenamic acid. Among them, niflumic acid, alongside its anti-inflammatory properties, has also documented the ability to inhibit cell proliferation and migration in various types of cancer [37]. There is also research on obtaining derivatives of niflumic acid with enhanced antitumor activity, such as its esters [38], polymeric silver (I) complexes based on niflumic acid [39], and Co(II) and Ni(II)-NA complexes [40].

Each year, the number of people diagnosed with cancer increases. Apart from genetic predispositions and viral or bacterial infections, there are immune system disorders that are responsible for the development of tumors. Meanwhile, chemotherapy treatment is often long-lasting and exhausting and causes side effects. Therefore, there is a need for safe methods to reduce these unwanted effects [41]. These include pharmacological pain therapies in cancer patients [42] so as to avoid the negative effects of opioids, generally used to treat moderate to severe cancer pain [43].

Despite the numerous studies on the biological activity of XN mentioned above, there are very limited studies exploring their effects on model membranes. For instance, the results of Arczewska and co-authors [44] showed that XN changed the physical properties of a model membrane (dry DPPC multibilayers) by influencing the molecular organization and structural properties in the polar part of the lipid bilayer. The authors concluded that XN is located in the interface region between the polar heads of lipids. Different conclusions were drawn by Wesołowska and co-authors [45], who showed that XH can penetrate into the hydrophobic region of the bilayer due to its linear shape. In contrast with the first group, these studies were conducted on fully hydrated DPPC membranes. Interestingly, to the best of our knowledge, no reports on the direct interactions of NA with model membranes have been published. The present work explains the synergistic effect of combined XA and NA on a model tumor cell membrane for the first time.

**Figure 1:**
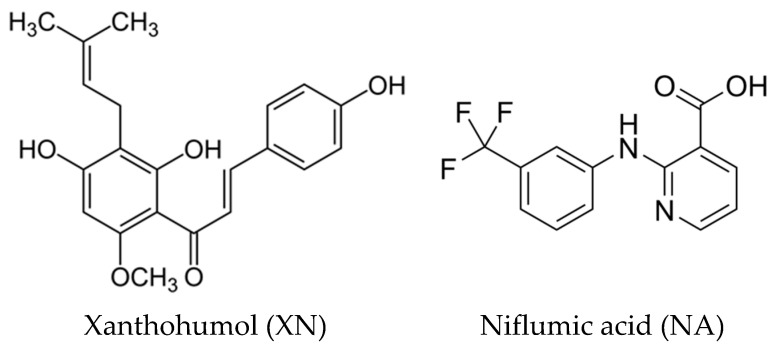
Chemical structures of tested compounds, XN and NA.

So as to support the development of new cancer strategies, the aim of this research was to evaluate the antiproliferative activity of known members of the NSAID group—niflumic acid (NA) in combination with the main prenyl hop chalcone xanthohumol (XN) (Figure 1)—against the cells of skin cancer (Merkel cell carcinoma (MCC13)) and glioblastoma (A172). The activity of this combination was compared with the activity of XN and NA applied separately. This study contributes to the current knowledge of new strategies for the prevention and treatment of cancers with the help of natural antioxidants belonging to flavonoids. In addition, we show the possibility of using difficult-to-dispose industrial waste, i.e., spent hops, as a source of valuable bioactive substances intended to be used for medical purposes.

## 2. Results

### 2.1. Antiproliferative Effects of Xanthohumol and Niflumic Acid on Cancer and Normal Cell Lines

The results of our study on the antiproliferative properties of the tested compounds are summarized in Table 1. Xanthohumol demonstrated high antiproliferative activity against all the tested cell lines. The lowest IC_50_ values, in the range from 12.3 ± 6.4 to 15.4 ± 7.9 μM, were noted for the A-172, 5637, A-431, and SK-MEL-3 cell lines. Higher IC_50_ values were observed for head and neck cancer cells (UM-SCC-17A) and Merkel cell carcinoma cells MCC-13, with values of 32.3 ± 9.8 and 23.4 ± 6.3 μM, respectively.

Over 10 times higher IC_50_ values compared with XN were noted for pure niflumic acid. For most of the tested cell lines, the values were in the range from 103.5 ± 17.1 μM (5637 cells) to 175.4 ± 6.9 μM (MCC13 cells). The lowest antiproliferative activity of niflumic acid was observed for UM-SCC-17A cells, with IC_50_ = 310.3 ± 32.6 μM.

**Scheme 1:**
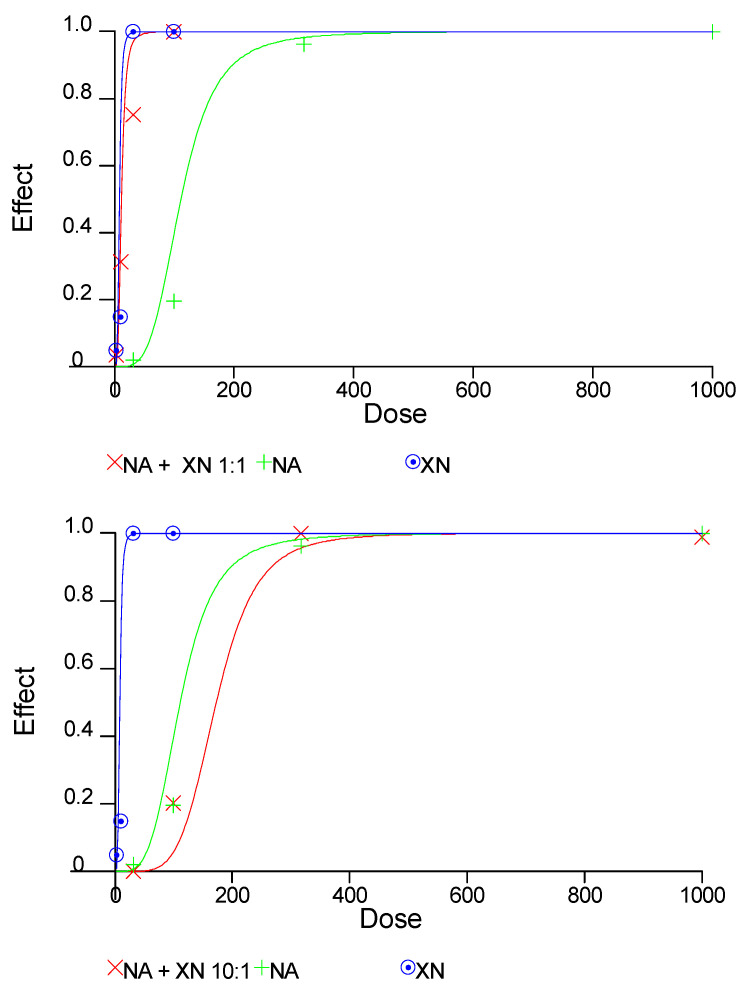
Dose effect curves of combined treatment with XN and NA on MCC13 cancer cells.

**Scheme 2:**
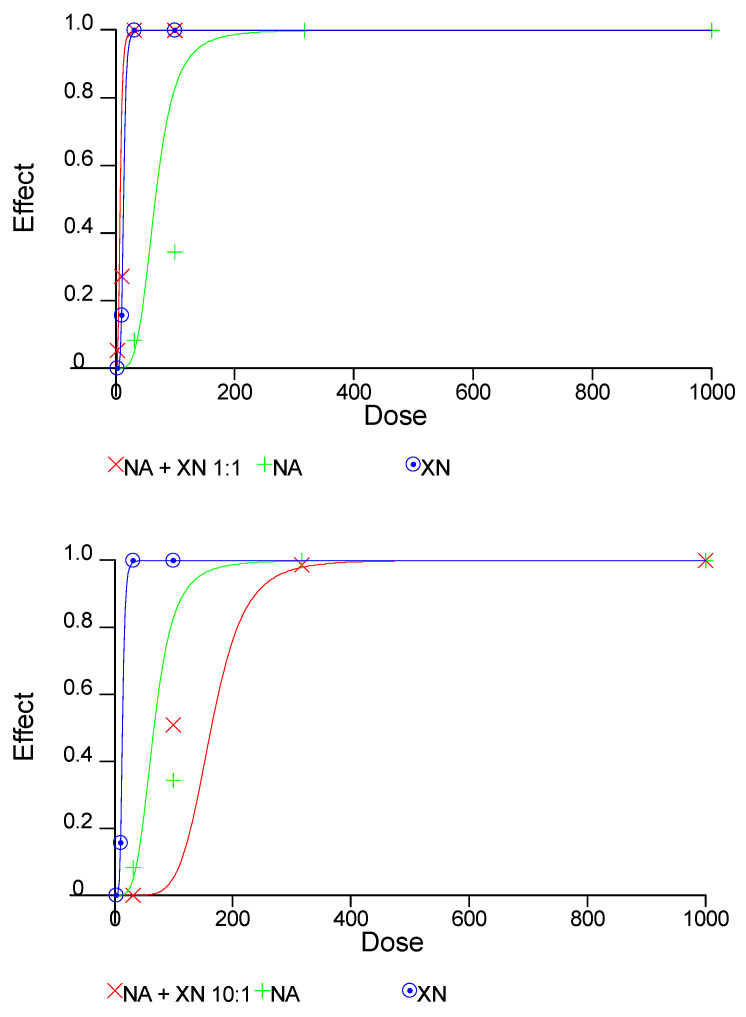
Dose effect curves of combined treatment with XN and NA on A172 cells.

The application of niflumic acid together with XN affected its antiproliferative activity against MCC13 and A172 cells; however, the results depended on the concentrations of the compounds and their ratio to each other (Scheme 1 and Scheme 2).

In the case of Merkel cell carcinoma cells (MCC-13), the synergistic effect of NA and XN was observed when low and equimolar doses of the two compounds were used (3.17 μM) (Table 2). The resulting combination index value (CI) was equal to 0.97. An even stronger effect was observed for an NA–XN ratio of 1000:100 μM (i.e., 317 and 31.7 μM, respectively), for which CI = 0.693. The other tested combinations resulted in an antagonistic effect, with the highest CI value of 12.475 for an NA–XN ratio of 10:1 μM (i.e., 31.7 and 3.17 μM, respectively).

Meanwhile, in the case of A-172 cells, the most beneficial effects were observed when the two tested compounds were used in an equimolar ratio, both at a concentration of 31.7 μM and 10 times lower, i.e., 3.17 μM (Table 3). The CI values calculated for these combinations were very similar: 0.523 and 0.530, respectively. The application of NA and XN in a ratio of 10:1 also gave an antagonistic effect.

### 2.2. Interaction with Model Membranes

**Figure 2:**
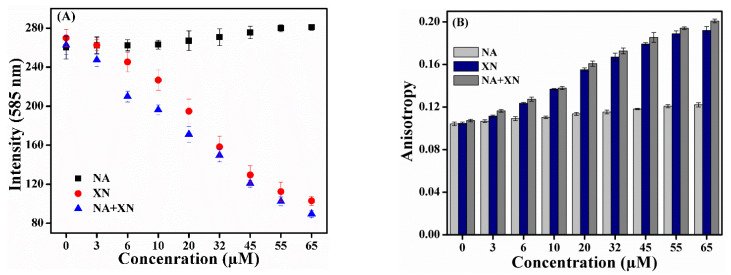
Effects of studied compounds on the (**A**) fluorescence intensity and (**B**) steady-state anisotropy of MC540 for model membranes at 37 °C. Values are means ± standard errors of the means (SEMs).

The fluorescence of MC540 is directly related to the order of lipids in the phospholipid bilayer and is highly dependent upon the polarity of the local environment [46,47,48,49]. In instances where membrane lipids exhibit a lower packing density, MC540 is parallel to and partitions into the phospholipid chains, resulting in an emission maximum at approximately 585–590 nm [45,50]. As displayed in Figure 2A, the presence of an increasing concentration of XN potently decreased the fluorescence intensity of MC540 compared with the control, which correlated with higher packing orders. In contrast, the fluorescence intensity of the probe was slightly higher in the presence of NA (~8.02%). This observation suggests that the organization of the lipids decreased, i.e., the lipid packing order was disturbed in the presence of NA. Interestingly, the combined effect of XN and NA (1:1) was more pronounced, indicating the greater rigidity of the model membranes due to synergistic effects. The observations from the fluorescence measurements were further supported by the steady-state anisotropy analysis. The rotational diffusion of the MC540 fluorescence anisotropy can be attributed to the fluidity of the surrounding environment. Molecules that restrict acyl chain motions are also thought to increase membrane rigidity and enhance fluorescence anisotropy [51,52]. The addition of XN resulted in increased membrane rigidity, while NA did not exert the same effect within the studied concentration range. However, the combination of XN and NA significantly enhanced the fluorescence anisotropy values, which, again, suggested synergistic rigidity effects on the MIMIC.

## 3. Discussion

Due to the chemoresistance of tumor cells to many drugs used in cancer therapy, there is a constant search for new strategies, aiming to improve the therapeutic effectiveness and alleviate the side effects of the treatments. One of these methods is using adjuvant substances of plant origin, including flavonoids, which have documented health-promoting properties and are used in combination with known and commonly used medicines.

Also, plant extracts containing mixtures of natural antioxidants are successively studied for their hepatoprotective activity in NSAID-induced liver injury [53]. It is also known that hop extracts (*Humulus lupulus* L.) have detoxification activity by reducing oxidative stress and suppressing liver inflammation [54]. Moreover, combination treatment with two or more active substances that have different mechanisms of action may change their properties and lead to enhanced synergistic activity. Examples include quercetin, which enhances the efficacy of prostate cancer treatment with docetaxel [55]; apigenin, which alleviates the doxorubicin resistance of breast cancer cells [56]; baicalein, which enhances the effects of docetaxel in the treatment of thyroid cancer [57]; and rutin, which demonstrates enhancing effects when used in combination with NSAIDs and paracetamol [58]. Other examples are bifunctional conjugates of indomethacin, an indole-acetic acid derivative used for the treatment of rheumatoid arthritis, with flavonoids such as naringenin or hesperetin. These conjugates cause weaker adverse effects of gastrointestinal origin than indomethacin alone while retaining high anti-inflammatory effectiveness, and, therefore, they can be used as safer NSAIDs [59]. Another study revealed that animals fed with naproxen combined with eicosapentaenoic acid (ω-3, EPA) exhibited a 95% reduction in the formation of colorectal cancer and a 98% reduction in tumor volume, which correlated with significantly reduced levels of proinflammatory ω-6 eicosanoids in the colonic mucosa [60].

One of the aims of targeted molecular therapy is to induce apoptosis in cancer cells through the inhibition of specific cell-signaling pathways. According to the literature, increased levels of apoptosis in cancer cells treated with niflumic acid (NA) may result from blocking Cl^−^ channels and the activation of K+ channels [61]. Meanwhile, Kim et al. [62] studied the mechanism of apoptosis induced by cyclosporin A in a human hepatoma cell line. The results showed that treatment with NA markedly prevented CsA-induced apoptosis in HepG2 cells. Combined treatment with the Cox-2 inhibitor niflumic acid and the PPARγ ligand ciglitazone induces ER stress/caspase-8-mediated apoptosis in human lung cancer cells.

It has also been reported that xanthohumol activated mitochondrial apoptosis in non-small-cell lung cancer (NSCLC) cells through the upregulation of PUMA (p53-upregulated modulator of apoptosis) expression [63]. What is more, XN caused an increase in the formation of early and late apoptotic cells, determined by annexin V staining and multicaspase assays in prostate cancer. A detailed analysis confirmed the activation of the proapoptotic proteins Bax and p53 in PC3 cells [64]. An inducing effect of XN on human prostate cancer cells was also confirmed by Deeb et al. [65]. XN markedly augmented TRAIL-mediated apoptosis and cytotoxicity in prostate cancer cells according to Szliszka et al.’s study [66]. An initial study on the impact of XN on lymphocytes from patients with B-chronic lymphocytic leukemia (B-CLL) showed that XN induced the dose-dependent killing of B-CLL cells, which was associated with annexin V positivity and, thus, suggested an apoptotic mechanism of this cell death [67]. Moreover, an inhibiting effect of XN on the proliferation of HCT 116-derived colon cancer cells (the IC50 decreased from 4.1 μM after 24 h of treatment to 3.6 and 2.6 μM after 48 and 72 h, respectively), as well as a downregulation of the expression of the anti-apoptotic Bcl-2 protein after a 48–72 h incubation of the cells with XN, was observed by Pan et al. [68]. The inhibition of human leukemia K562 cell viability associated with the induction of apoptosis suggests that XN may have potential clinical applications in the treatment of Bcr-Abl(+) myeloid leukemia [69].

The anti-inflammatory effects of polymethoxylated flavones from citrus fruits in combination with hop prenylflavonoids (in the form of mixtures) were confirmed in a study on Caco-2 cells [70]. Meanwhile, Ho et al. [71] demonstrated that the effectiveness of the anticancer drug temozolomide in the treatment of glioblastoma may be enhanced with the help of xanthohumol. Another research team employed statins (simvastatin and mevastatin), drugs used for the treatment of hypercholesterolemia, in combination with selected hop flavonoids, 6-prenylnaringenin, 8-prenylnaringenin, and xanthohumol, as an effective method of increasing the efficacy and safety of cancer therapy [72]. Moreover, there is proof of the synergistic effect of aminochalcones and doxorubicin in the treatment of osteosarcoma [73] and also of the synergistic enhancement of the radiosensitivity of cancer cells by prenylflavonoids, like icaritin, and by hydroxychalcones without prenyl groups, like butein [74,75].

The available literature suggests that cancer cell membranes have higher fluidity compared with normal cells [76,77], and, thus, certain compounds may exhibit anticancer activity by showing a rigidifying effect on membranes. For instance, dietary flavonoids can stiffen membranes, which is one of the main mechanisms behind their anticancer properties, with the impact depending on the dosage [78,79].

**Figure 3:**
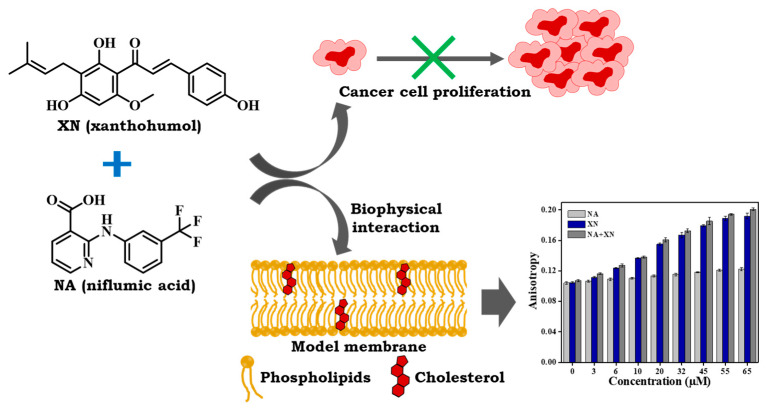
Scheme summarizing this study.

In our study, we confirmed the synergistic effect of two active substances, xanthohumol and niflumic acid, applied together, on Merkel cell carcinoma and glioblastoma cells (Figure 3).

Summarizing the facts, it is reasonable to believe that XN, both alone and in combination with NA, exhibited its anticancer activity by rigidifying the cancer cell membrane. Interestingly, our research demonstrates the synergistic effects of XN and NA on membrane rigidity, which could account for the enhanced inhibition of proliferation for the combination of XN and NA in the studied cancer cell lines. To the best of our knowledge, this is the first research of this kind, and the results indicate that anti-inflammatory drugs combined with natural prenylchalcones isolated from hops may be used in cancer treatments in the future. This will be possible after further, more detailed research on the effects of this combination and similar multicomponent systems on the human body in comparison with other NSAIDs in order to find an optimal therapeutic dose and eliminate possible side effects. For therapeutic applications, it is also important to establish the molecular mechanisms of action of such binary formulations based on niflumic acid and its derivatives combined with xanthohumol and other prenylflavonoids. Future studies should also involve a wider range of cell lines derived from various tissues and organs.

## 4. Materials and Methods

### 4.1. Materials

POPC, POPE, SOPS, cholesterol, and phosphate buffer (PBS) were purchased from Sigma-Aldrich (St. Louis, MO, USA). Ethanol and chloroform were bought from POCh (Gliwice, Poland). Merocyanine 540 (MC540) was purchased from Sigma Aldrich (Steinheim, Germany).

Xanthohumol (XN) (3′-(3,3-dimethyl allyl)-2′,4′,4-trihydroxy-6′-methoxychalcone) was isolated from spent hops obtained from the New Chemical Syntheses Institute in Puławy, Poland. Specifically, the material used for xanthohumol isolation was the byproduct of the hops’ industrial extraction with supercritical CO_2_. Gradient-grade purity methanol was purchased from Merck (Darmstadt, Germany). All the other reagents, unless otherwise specified, were purchased from Sigma-Aldrich (St. Louis, MO, USA) and were of analytical grade.

### 4.2. General Procedures

Analytical thin-layer chromatography, preparative column chromatography, NMR spectra, and HPLC analysis were carried out according to previously described methods [80]. The ISIS/Draw version 2.3 software was used to draw the molecular structures.

The content of XN was detected at 368 nm. The spectral data of XN were consistent with those published before [81].

### 4.3. Cell Cultures and Laboratory Assays

#### 4.3.1. Cell Culture Conditions

Six human cancer cell lines and one murine normal cell line were used to evaluate the antiproliferative activity of NA and XN: the 5637 human urinary bladder, A-431 human epidermoid carcinoma, UM-SCC-17A human head and neck squamous carcinoma, SK-MEL-3 human melanoma, MCC-13 Merkel cancer, A172 glioblastoma, and BALB/3T3 clone A31 normal murine embryonic fibroblast cell lines. The UM-SCC-17A and MCC13 cell lines were purchased from the European Collection of Authenticated Cell Cultures (ECACC, Salisbury, UK) by M. Stompor-Gorący (the University of Rzeszów, Rzeszów, Poland). The A-431, SK-MEL-3, A172, and BALB/3T3 clone A31 cell lines were purchased from the American Type Culture Collection (ATCC, Manassas, VA, USA). The 5637 cell line was purchased from the Riken BRC Cell Bank. All of the cell lines were maintained at the Institute of Immunology and Experimental Therapy (IIET), Wroclaw, Poland. The 5635 cells were cultured in a mixture of Roswell Park Memorial Institute (RPMI) 1640 (medium (IIET, Wroclaw, Poland), supplemented with HyClone 10% fetal bovine serum (GE Healthcare) and 2 mM of L-glutamine (Sigma-Aldrich, Steinheim, Germany). The UM-SCC-17A cells were cultured in Dulbecco’s medium (IIET, Wroclaw, Poland), supplemented with 15% fetal bovine serum and 2 mM of L-glutamine (Sigma-Aldrich, Steinheim, Germany). The SK-MEL-3 cells were cultured in McCoy 5A medium (Corning, Corning, NY, USA) with L-glutamine, supplemented with HyClone 15% fetal bovine serum (GE Healthcare, Chicago, IL, USA). The MCC13 cells were cultured with RPMI with 25 mM of HEPES medium, supplemented with HyClone 15% fetal bovine serum (GE Healthcare) and 2 mM of L-glutamine (Sigma-Aldrich, Steinheim, Germany). The A-431, A172, and BALB/3T3 fibroblasts were cultured in Dulbecco’s medium (Gibco, Grand Island, NY, USA), supplemented with 10% fetal bovine serum (GE Healthcare) and 2 mM of L-glutamine (Sigma-Aldrich, Steinheim, Germany). All culture media contained the following antibiotics: 100 U/mL of penicillin (Polfa Tarchomin, Poland) and 0.1 mg/mL of streptomycin (Sigma Aldrich, Steinheim, Germany). All cell lines were cultured during the entire experiment in a humid atmosphere at 37 °C and 5% CO_2_.

Twenty-four hours before adding the tested compounds, all the cells were seeded in 384-well plates (Greiner Bio-One, Kremsmünster, Austria) in appropriate media with (5 × 10^3^) − (5 × 10^4^) cells per well. All the cell lines were exposed to each of the tested agents at various concentrations, ranging from 100 to 0.0316 μM, for 72 h. The cells were also exposed to the reference drug, cisplatin (ACCORD, Warsaw, Poland). Additionally, all the cell lines were exposed to dimethyl sulfoxide (DMSO) (the solvent used for the solutions of the tested compounds) (POCh, Poland) at concentrations corresponding to those present in the tested agent dilutions. After 72 h, the sulforhodamine B (SRB) assay was performed.

#### 4.3.2. SRB Assay

The SRB assay was performed according to Krzywik et al., with modifications [82]. After 72 h of incubation with the tested compounds, the cells were fixed in situ by gently adding cold 25% trichloroacetic acid (TCA) (POCh, Poland) and were incubated at room temperature for 1 h. Then, the wells were washed four times with water and dried. Next, a 0.1% solution of sulforhodamine B (Sigma-Aldrich, Steinheim, Germany) in 1% acetic acid (POCh, Poland) was added to each well, and the plates were incubated at room temperature for 0.5 h. After the incubation time, the unbound dye was removed by washing the plates four times with 1% acetic acid, whereas the stain bound to cells was solubilized with 10 mM of Tris base (Sigma-Aldrich, Steinheim, Germany). The absorbance of each solution was read with a Synergy H4 Hybrid Multi-Mode Microplate Reader (BioTek Instruments, Winooski, VT, USA) at a wavelength of 540 nm. The results were calculated as 50% inhibitory concentration (IC_50_) values, namely, the dose of a tested compound inhibiting the proliferation of the cells by 50% compared with the untreated control cells, in the Prolab-3 system based on the Cheburator 0.4 software using the two-point method [83]. The compounds at each concentration were tested in triplicate in a single experiment, and each experiment was repeated at least three times independently.

#### 4.3.3. Combination of XN with NA

Cells (MCC13 or A172) were seeded in 384-well plates (Corning) at a density of 4 × 10^4^ cells/well in 50 μL of the culture medium without FBS and antibiotics. After 24 h of incubation in standard conditions, the cells were treated with four solutions of a mixture of the compounds NA and XN in two ratios: 1:1 (starting from 100 μM) and 10:1 (starting from 1000 µM of NA). Non-treated cells as well as cells treated with single compounds served as controls. After an additional 72 h of incubation, the sulforhodamine B (SRB) assay was performed as described earlier by Skehan et al. [84]. The absorbance of the samples was read using the Synergy H4 Hybrid Reader (BioTek Instruments, Winooski, VT, USA) at a 540 nm wavelength. To define the interactions between NA and XN, the results of the SRB assay were analyzed using the CalcuSyn program (Biosoft, Cambridge, UK). Based on the method described by Chou and Talalay [85,86], the combination index (CI) was calculated. Values of CI <1, =1, and >1 indicated synergism, an additive effect, and antagonism, respectively.

### 4.4. Preparation of MIMIC

The model membrane that mimicked the composition of tumor membranes (48 mol% POPC, 24 mol% POPE, 8 mol% SOPS, and 20 mol% cholesterol) was prepared according to the reported procedure in [87,88]. The total lipid concentration in the working solution was 0.1 mg/mL. To obtain small unilamellar vesicles (SUVs), multilamellar vesicles were extruded 10 times through nucleopore polycarbonate filters (Whatman, Maidstone, UK) with a pore size of 100 nm using a Thermobarrel Extruder (10 mL Lipex extruder, Northern Lipids, Canada). To determine the effects of the compounds on the model membrane, especially to monitor the molecular packing of the phospholipids in the membrane, we used the fluorescent probe MC540.

MC540 (2 μM) was added to the prepared lipids, and the solution was incubated in the dark for at least 2 h followed by the addition of the studied compounds in the concentration range of 3–65 µM. The steady-state fluorescence intensity was recorded with a CARY Eclipse fluorimeter (Varian, San Diego, CA, USA) equipped with a DBS Peltier temperature controller (temperature accuracy: ±0.1 °C). Steady-state anisotropy measurements were obtained with the same instrument using the MC540 probe (with excitation at 540 nm and emission at 585 nm). The fluorescence anisotropy values (*r*) were calculated using the following equation [89]:

(1) $$r=\frac{I_{VV}-GI_{VH}}{I_{VV}+2GI_{VH}}$$

where *I_VV_* and *I_VH_* denote the fluorescence intensities observed in directions parallel and perpendicular to the polarization direction of the exciting wave, respectively. *G* represents an apparatus constant that is contingent upon the emission wavelength.

Studies on the interactions of the compounds with the model membranes were performed in three independent experiments. All data are presented as means with standard deviations (SDs).

## 5. Conclusions

In this study, we determined the antiproliferative activity of xanthohumol and niflumic acid applied separately against the following cell lines: 5637, A-431, UM-SCC-17A, SK-MEL-3, MCC-13, A172, and BALB/3T3 clone A31. After a 72 h exposure to the tested compounds, the IC_50_ for XN was in the range from 12.3 ± 6.4 to 32.3 ± 9.8 μM, whereas for NA, it was from 103.5 ± 17.1 to 310.3 ± 32.6 μM.

For the first time, we also noticed that the combination of xanthohumol and niflumic acid inhibited cell viability more effectively than xanthohumol and niflumic acid alone in skin (MCC13) and glioblastoma (A172) cancer cells. The combination potentiated inhibition proliferation in both cell lines in a dose-dependent manner. In addition, in order to understand the mechanism underlying the anticancer properties of the tested compounds, their effects on the biophysical parameters of the model membrane were evaluated. These studies showed a synergistic effect on membrane stiffness with a mixture of XN and NA, which may explain the observed increase in anticancer activity for the combination of XN and NA.

However, to sustain the idea of the role of NSAID–flavonoid combinations as anticancer agents, further studies should be performed to fully define the action of such combinations and the underlying mechanisms in vivo. Based on our results, we are of the opinion that this combination offers a promising approach to cancer therapy in the future.

## Figures and Tables

**Table 1:** Antiproliferative effects of XN and NA on selected cell lines.

Compound	IC_50_ [μM]						
	5637 Urinary Bladder Carcinoma	A-431 Epidermoid Carcinoma	UM-SCC-17A Head and Neck Squamous Carcinoma	SK-MEL-3 Melanoma	MCC-13 Merkel Cancer	A172 Glioblastoma	BALB/3T3 Clone A31 Mouse Normal Fibroblast
NA	103.5 ± 17.1	153.3 ± 43.1	310.3 ± 32.6	167.1 ± 4.4	175.4 ± 6.9	143.2 ± 6.5	158.8 ± 33.0
XN	12.3 ± 6.4	14.8 ± 2.9	32.3 ± 9.8	15.4 ± 7.9	23.4 ± 6.3	14.4 ± 4.7	12.3 ± 5.5
Cisplatin^c^	0.5 ± 0.2	1.2 ± 0.4	5.2 ± 1.3	4.2 ± 1.4	2.2 ± 1.0	7.1 ± 2.3	0.5 ± 0.2
c—IC_50_ measured in μg/mL							

**Table 2:** The antiproliferative effects of combined treatment with XN and NA on MCC13 cancer cells.

Compound (Concentration Ratio)	IC_50_ ± SD	Concentration [μM]/CI (Combination Index) *				
NA + XN (1:1)	21.7 ± 15.6	NA	100	31.7	10	3.17
		XN	100	31.7	10	3.17
			**1.465**	**3.328**	**1.665**	**0.970**
NA + XN (10:1)	152.2 ± 19.6	NA	1000	317	100	31.7
		XN	100	31.7	10	3.17
			**7.401**	**0.693**	**3.085**	**12.475**
NA	160.4 ± 19.4					
XN	16.0 ± 2.0					

* Meaning of CI values calculated by CalcuSyn Version 2.1 software (Biosoft, Cambridge, UK): CI < 1—synergism, CI = 1—additive effect, and CI > 1—antagonism.

**Table 3:** The antiproliferative effects of combined treatment with XN and NA on A172 cells.

Compound (Concentration Ratio)	IC_50_ ± SD	Concentration [μM]/CI (Combination Index)				
NA + XN (1:1)	14.2 ± 1.5	NA	100	31.7	10	3.17
		XN	100	31.7	10	3.17
			**1.648**	**0.523**	**1.133**	**0.530**
NA + XN (10:1)	99.2 ± 11.2	NA	1000	317	100	31.7
		XN	100	31.7	10	3.17
			**2.353**	**2.403**	**2.279**	**21.526**
NA	131.4 ± 11.7					
XN	15.8 ± 1.3					

## Data Availability

Data is contained within the article.
